# Risk factors for postoperative recurrence of desmoid tumors: a retrospective cohort analysis

**DOI:** 10.3389/fonc.2025.1677325

**Published:** 2026-01-16

**Authors:** Yue Peng, Shiyao Liu, Yi Li, Xiaoyu Chen, Yuanxin Hu, Guobo Du

**Affiliations:** 1 Department of Oncology, Affiliated Hospital of North Sichuan Medical College, Nanchong, Sichuan, China; 2 Department of Clinical Medicine, North Sichuan Medical College, Nanchong, China

**Keywords:** desmoid tumors, recurrence-free survival, retrospective cohort analysis, risk stratification, surgical management

## Abstract

**Objective:**

Desmoid tumors (DTs) are rare soft-tissue neoplasms characterized by local invasiveness and high recurrence potential. Due to heterogeneous clinical behavior and unresolved controversies regarding postoperative recurrence risk factors, optimal management remains challenging. This study aimed to identify predictors of recurrence-free survival (RFS) after grossly complete recection.

**Methods:**

We retrospectively analyzed 81 DT patients undergoing resection at our institution (September 2014–December 2024). Clinicopathological variables were assessed using Cox regression for recurrence risk, with RFS compared via Kaplan-Meier analysis.

**Results:**

The median follow-up was 55 months (range: 4–127 months). The 1-year, 3-year, and 5-year RFS rates for the entire cohort were 89.8% (95% CI: 83.1-96.5), 73.8% (95% CI: 63.6-84.0), and 65.5% (95% CI: 53.5-77.5), respectively. Univariate Cox regression analysis showed that larger tumor size (per 1 cm increase: HR = 1.271, *p* < 0.001), recurrent disease status (HR = 2.741, *p* = 0.027), and tumor location on an extremity (HR = 2.62, *p* = 0.021) were significantly associated with poorer prognosis. To mitigate the risk of overfitting, the subsequent multivariate analysis was limited to two variables. This model identified both tumor size (HR = 1.283, *p* < 0.001) and extremity location (HR = 2.899, *p* = 0.011) as independent risk factors. The robustness of these findings was further confirmed using a Bonferroni correction (adjusted significance level α = 0.025).

**Conclusion:**

In this cohort, tumor size and extremity location were identified as independent predictors of worse recurrence-free survival in patients with desmoid tumors following macroscopic complete resection. These robust factors may aid in postoperative risk stratification and inform patient counseling.

## 1 Introduction

Desmoid tumors (DTs) are rare, fibroblastic-origin soft tissue neoplasms characterized by locally aggressive growth. Although associated with low mortality, the substantial psychological and financial burdens imposed by this disease, as well as its detrimental effects on quality of life, must not be overlooked (1). The core clinical feature of DTs lies in their unpredictable natural history, manifesting as spontaneous stabilization/regression, progression, or recurrence, this heterogeneity poses significant challenges for clinical decision-making (2, 3). Based on natural history studies of DTs and evidence supporting the efficacy of non-surgical management, a therapeutic paradigm shift has occurred in recent years (4–6). As a result, international guidelines and consensus statements recommend that active surveillance be established as the standard first-line management strategy for asymptomatic, treatment-naïve DTs, while surgical and other invasive interventions should be reserved for patients with progressive disease or symptomatic presentations (2, 7). This conservative “watch-and-wait” approach reduces the risk of functional impairment associated with overtreatment, thereby advancing DTs management into an era of individualized care grounded in multidisciplinary team collaboration. For patients with progressive or symptomatic disease, surgery retains therapeutic value; however, specific risk factors for postoperative recurrence—including tumor location, size, and margin status—have not been fully established, and controversies regarding these factors persist (8–11). The recurrence risk of DTs exhibits marked heterogeneity. Consequently, the precise identification of key predictors for postoperative recurrence is critical for developing individualized therapeutic approaches and improving clinical outcomes. Through a single-center retrospective cohort study of surgically managed DTs patients, this study aims to validate independent prognostic factors for RFS, thereby providing an evidence-based foundation for risk-stratified management of surgical patients.

## 2 Materials and methods

### 2.1 Patient cohort

This was a retrospective cohort study. We included patients with sporadic DTs who underwent grossly complete resection at the Affiliated Hospital of North Sichuan Medical College between September 2014 and December 2024. The inclusion criteria were: (1) histopathological confirmation of DT, and (2) having undergone grossly complete resection. Exclusion criteria comprised: (1) patients with familial adenomatous polyposis (n = 3), due to distinct pathogenesis and recurrence risk profiles (12–15); (2) patients who did not undergo curative surgery (such cases were excluded during the initial search); and (3) patients lost to follow-up or with incomplete outcome data (n = 7). Of the initially identified 91 patients, 81 met all criteria and were included in the final analysis. Tumor were categorized by anatomical site into “extremity” and “non-extremity” (head/neck, trunk, or abdomen) for survival analysis. The overall cohort (N = 81) included all patients who received grossly complete resection at our institution, consisting of 67 with primary tumors and 14 who were referred for surgery after recurrence following initial treatment elsewhere.

The study protocol was approved by the Institutional Review Board of the Affiliated Hospital of North Sichuan Medical College (Approval No. 2025ER290-1). The requirement for informed consent was waived due to the retrospective nature of the study and the use of anonymized data.

### 2.2 Data collection

Clinical data for all patients were acquired through inpatient records, outpatient documentation, and telephone follow-up, encompassing demographic characteristics (sex, age), tumor features (location, maximum diameter, adjacency to major vessels/nerves, immunohistochemistry profiles), treatment details, tumor-related history (prior surgery or trauma in primary tumor region), recurrence status during follow-up, last follow-up date, and vital status. RFS was defined as follows: primary cohort patients: time from initial surgery to radiological/pathological confirmation of recurrence; the duration from surgery date to pathologically or radiologically confirmed recurrence. Tumor size was determined by the maximum diameter measured on pathological reports.

### 2.3 Patient follow-up and study outcome definitions

Patient follow-up and surveillance were conducted in accordance with the standard clinical practice during the study period. Postoperatively, routine imaging was recommended every 6 to 12 months for the first 2 to 3 years, with intervals potentially extended to annually thereafter for the early detection of local recurrence. Surveillance primarily involved contrast-enhanced computed tomography (CT) and/or magnetic resonance imaging (MRI), with the specific modality chosen based on the primary tumor site and clinical judgment. The primary outcome of this study was postoperative recurrence, defined as the emergence of a new lesion in the original surgical field or adjacent area, confirmed by imaging (CT or MRI) or by pathological verification upon re-operation. To ensure data consistency, all available imaging reports and clinical records for the included patients were systematically collected and reviewed.

### 2.4 Statistical analysis

Categorical variables are presented as frequencies (percentages). All statistical analyses were performed using SPSS software version 27.0 (IBM Corp., Armonk, NY). Pearson’s chi-square test was used to compare categorical variables (e.g., clinicopathological characteristics) between groups. RFS was defined as the interval from the date of initial surgery to the date of radiologically or pathologically confirmed recurrence for primary cohort patients. RFS was estimated using the Kaplan-Meier method, and differences in RFS between groups were compared using the log-rank test.

Prognostic factor analysis proceeded as follows. First, univariable Cox proportional hazards regression was performed for all candidate variables to calculate crude hazard ratios with 95% confidence intervals. To reduce the risk of omitting important variables in the limited sample (type II error), a significance level of *p* < 0.10 was set for entry into the multivariable analysis. For continuous variables with *p* < 0.10 in the univariable analysis, the linearity assumption of their association with the log hazard ratio was further tested. This was done using restricted cubic splines with three knots placed at the 10th, 50th, and 90th percentiles of the variable. The significance of the nonlinear component was assessed by a Wald test. If the nonlinear component was not statistically significant, the variable was treated as a continuous linear term in subsequent models; otherwise, spline functions or variable transformation were considered. Continuous variables that passed the linearity test, along with categorical variables with *p* < 0.10 in the univariable analysis, were entered into a multivariable Cox proportional hazards regression model. Given the limited number of recurrence events, a maximum of two covariates were allowed in the multivariable model to strictly control the risk of overfitting. Final variable selection was based on statistical significance and clinical relevance.

Recognizing that multiple comparisons in the multivariable model could increase the risk of type I error, we planned a sensitivity analysis using the Bonferroni correction: the conventional significance level (α = 0.05) was divided by the number of variables retained in the final model to obtain an adjusted threshold. Both unadjusted *p*-values and inferences based on the Bonferroni-corrected threshold are reported to assess the robustness of the findings. All tests were two-sided, and a *p*-value < 0.05 was considered statistically significant unless otherwise specified.

## 3 Result

### 3.1 Baseline clinicopathological characteristics

To compare clinicopathological characteristics between abdominal and extra-abdominal fibromatosis patients, we retrospectively analyzed 81 cases (median age: 35 years; range: 1–85 years). As shown in **Table 1**, females constituted 66.7% (54/81) of the cohort, with 50.6% (41/81) being women of reproductive age. Solitary lesions were present in 96.3% (78/81) of patients. Tumor distribution analysis revealed 33.3% (27/81) abdominal and 66.7% (54/81) extra-abdominal tumors. Among abdominal tumors, 59.3% (16/27) originated from the abdominal wall and 40.7% (11/27) were intra-abdominal. Extra-abdominal tumors primarily involved the extremities (51.9%, 28/54), trunk (31.5%, 17/54), and head/neck (16.7%, 9/54). Comparative analysis demonstrated significantly higher proportions of tumors adjacent to vascular/neural structures (48.1% *vs*. 16.7%, *p* = 0.003) and prior surgical history at the tumor location (44.4% *vs*. 22.2%, *p* = 0.039) in abdominal tumors versus extra-abdominal tumors. Recurrences originated predominantly from extra-abdominal sites (24.1% *vs*. 3.7% of tumors, *p* = 0.048), and the recurrence rate was significantly lower for abdominal tumors than for extra-abdominal tumors (7.4% *vs*. 38.9%, *p* = 0.003), suggesting reduced recurrence risk for abdominal tumors.

**Table 1:** Comparison of clinicopathological characteristics between abdominal and extra-abdominal desmoid tumors^#^.

Characteristic	Total	Abdominal	Extra-abdominal	χ²	*P* value
	(n=81)	(n=27)	(n=54)		
Gender					
Male	27 (33.3)	11 (40.7)	16 (29.6)	1	0.317
Female	54 (66.7)	16 (59.3)	38 (70.4)		
Age					
<40 years	49 (60.5)	18 (66.7)	31 (57.4)	0.646	0.422
≥40 years	32 (39.5)	9 (33.3)	23 (42.6)		
Symptoms					
Asymptomatic	49 (60.5)	17 (63.0)	32 (59.3)	0.103	0.748
Symptomatic	32 (39.5)	10 (37.0)	22 (40.7)		
Admission status					
Primary	67 (82.7)	26 (96.3)	41 (75.9)	3.897	0.048^*^
Recurrent	14 (17.3)	1 (3.7)	13 (24.1)		
Medical history					
Positive	24 (29.6)	12 (44.4)	12 (22.2)	4.263	0.039^*^
Negative	57 (70.4)	15 (55.6)	42 (77.8)		
Maximum diameter					
<7cm	36 (44.4)	10 (37.0)	26 (48.1)	0.9	0.343
≥7cm	45 (55.6)	17 (63.0)	28 (51.9)		
Adjacent to vessels/nerves					
No	59 (72.8)	14 (51.9)	45 (83.3)	9.017	0.003^*^
Yes	22 (27.2)	13 (48.1)	9 (16.7)		
Single/Multiple					
Solitary	78 (96.3)	26 (96.3)	52 (96.3)	N/A	N/A
Multiple	3 (3.7)	1 (3.7)	2 (3.7)		
Treatment					
Surgery alone	68 (84.0)	25 (92.6)	43 (79.6)	N/A	N/A
Surgery + radiotherapy	13 (16.0)	2 (7.4)	11 (20.4)		
Surgical repair					
Primary closure	66 (81.5)	19 (70.4)	47 (87.0)	3.314	0.069
Complex repair^**^	15 (18.5)	8 (29.6)	7 (13.0)		
Mesh reconstruction	7 (46.7)	6 (75.0)	1 (14.3)		
Flap transplantation	2 (13.3)	0	2 (28.6)		
Flap repair	4 (26.7)	0	4 (57.1)		
Stoma creation	2 (13.3)	2 (25.0)	0		
Recurrence status					
Censored	58 (71.6)	25 (92.6)	33 (61.1)	8.774	0.003^*^
Recurred	23 (28.4)	2 (7.4)	21 (38.9)		
Ki67 index					
≥5%	35 (55.6)	14 (58.3)	21 (53.8)	0.121	0.728
<5%	28 (44.4)	10 (41.7)	18 (46.2)		
CD34					
Negative	51 (85.0)	18 (81.8)	33 (86.8)	0.023	0.881
Positive	9 (15.0)	4 (18.2)	5 (13.2)		
S100					
Negative	58 (93.5)	22 (91.7)	36 (94.7)	N/A	N/A
Positive	4 (6.5)	2 (8.3)	2 (5.3)		
Desmin					
Negative	24 (47.1)	8 (44.4)	16 (48.5)	0.076	0.782
Positive	27 (52.9)	10 (55.6)	17 (51.5)		
SMA					
Negative	17 (28.8)	7 (30.4)	10 (27.8)	0.048	0.826
Positive	42 (71.2)	16 (69.6)	26 (72.2)		
β-catenin					
Negative	3 (5.3)	0 (0.0)	3 (8.6)	N/A	N/A
Positive	54 (94.7)	22 (100.0)	32 (91.4)		

# Data are presented as number of patients (%), χ² test was used for comparison between groups.

* Values in bold denote statistically significant differences (p < 0.05).

** For the subcategories of surgical repair, percentages are calculated based on the number of patients who underwent complex repair in each group (abdominal group: n=8; extra-abdominal group: n=7).

### 3.2 Treatment modalities and surgical outcomes

All 81 patients underwent macroscopically complete resection. Postoperatively, 16% (13/81) received radiotherapy and 1.2% (1/81) received chemotherapy. Complex reconstructive procedures were required in 18.5% (15/81) of cases, including synthetic mesh reconstruction (46.7%, 7/15); local flap repair (26.7%, 4/15); skin grafting (13.3%, 2/15); and single-cavity ostomy (13.3%, 2/15). This indicates that additional interventions were necessary to optimize functional and aesthetic outcomes in a subset of patients. Notably, when comparing tumor groups, complex reconstructive procedures were more frequently required in the abdominal tumor group than in the extra-abdominal group (29.6% (8/27) *vs*. 13.0% (7/54)), though this difference did not reach statistical significance (*p* = 0.069).

### 3.3 Prognostic analysis of the entire cohort

**Figure 1:**
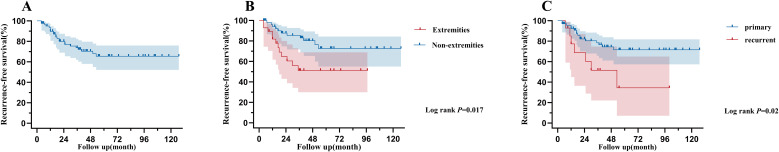
Kaplan-Meier curves for recurrence-free survival (RFS). **(A)** RFS in the overall cohort. **(B)** RFS stratified by tumor location (Extremities *vs*. Non-extremities; Log-rank *p* = 0.017). **(C)** RFS stratified by tumor status (Primary *vs*. Recurrent; Log-rank *p* = 0.02). All panels: X-axis, follow-up time (months); Y-axis, recurrence-free survival (%).

As of July 2025, the median follow-up time for this cohort was 55 months, calculated using the reverse Kaplan-Meier method, with a range of 4–127 months. No deaths occurred by the last follow-up. Tumor recurrence was observed in 23 patients (28.4%, 23/81; **Figure 1A**). The estimated 1-, 3-, and 5-year RFS rates were 89.8% (95% CI: 83.1–96.5), 73.8% (95% CI: 63.6–84.0), and 65.5% (95% CI: 53.5–77.5), respectively. Recurrence risk was higher in the early postoperative period and stabilized beyond 60 months of follow-up.

We first assessed the prognostic impact of tumor anatomical site. Based on clinical practice, sites were initially categorized into three groups: head/neck & trunk (n = 26), extremity (n = 28), and abdominal (n = 27). Kaplan-Meier curves (see **Supplementary Figure S1**) indicated that patients with extremity tumors had the lowest recurrence-free survival (RFS). However, only 2 recurrence events occurred in the abdominal group during the entire follow-up period. The limited sample size resulted in unstable survival estimates for this subgroup and precluded valid statistical comparison with the other two groups. To ensure robustness and statistical power in subsequent regression analyses, tumor site was consolidated into a binary variable for formal analysis: extremity (n = 28) *vs*. non-extremity (combining head/neck & trunk and abdominal sites, n=53). Based on this classification, survival analysis revealed that patients with extremity tumors had significantly worse RFS than those with non-extremity tumors (log-rank *p* = 0.017; **Figure 1B**). Furthermore, analysis stratified by disease status at treatment (primary *vs*. recurrent) showed that patients undergoing surgery for recurrent disease (n = 14) had significantly worse RFS than those with primary tumors (n = 67) (log-rank P = 0.02; **Figure 1C**). The recurrence rate was 23.9% (16/67) in primary patients and 50.0% (7/14) in previously recurrent patients. The RFS curves demonstrated a higher risk of recurrence in the early postoperative period, which later plateaued: the estimated RFS rate was approximately 92.3% at 12 months (1 year) and declined to about 76.5% at 36 months (3 years). Specifically, the recurrence rate was 42.9% (12/28) for extremity tumors and 20.8% (11/53) for non-extremity tumors. These findings suggest that tumor site (extremity *vs*. non-extremity) and disease status at treatment (primary *vs*. recurrent) may be important clinical factors influencing RFS.

**Figure 2:**
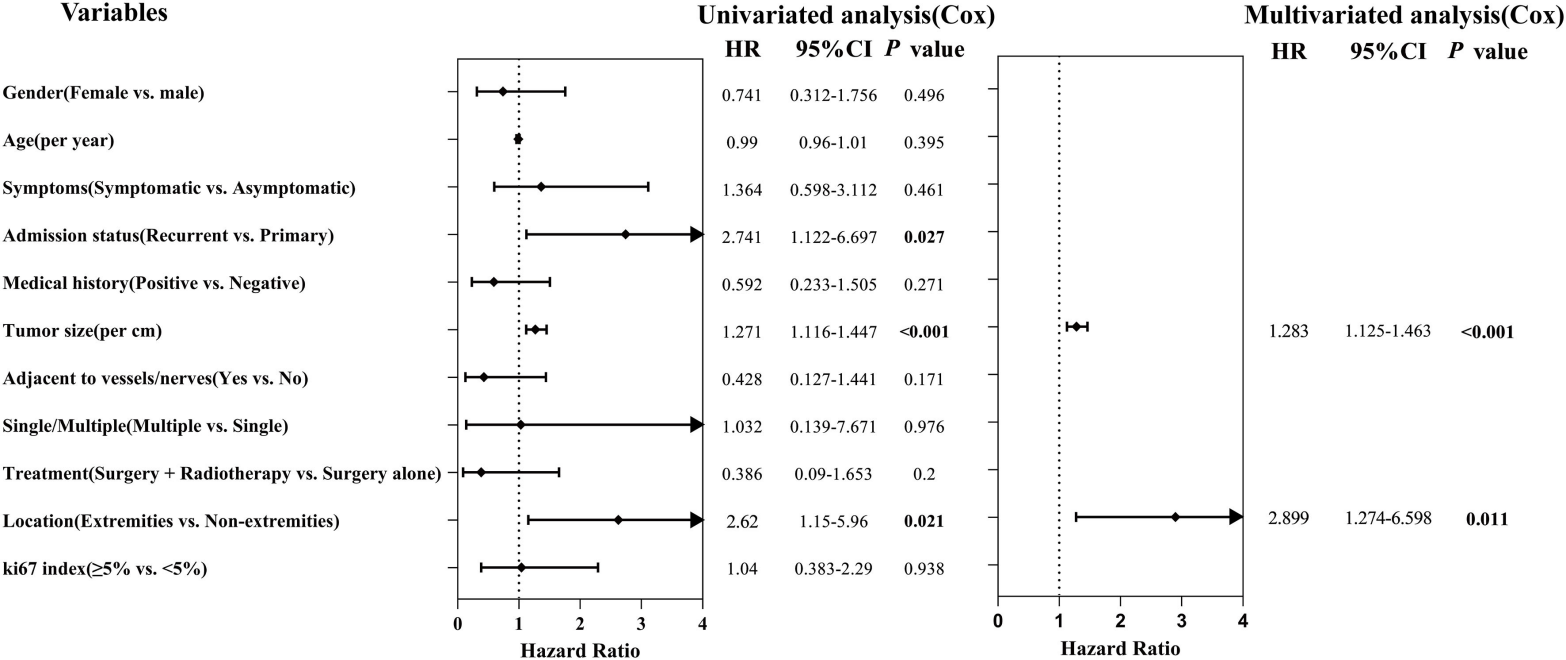
Forest plot of Cox regression analysis for RFS predictors. Multivariate model identified tumor size (per 1cm, HR = 1.283, *p* < 0.001) and extremity tumors (HR = 2.899, *p* = 0.011) as independent risk factors.

Univariable Cox regression analysis identified three prognostic factors potentially associated with postoperative recurrence risk (*p* < 0.10) (**Figure 2**). Specifically, each 1-centimeter increase in the maximum tumor diameter was associated with a 27.1% increase in recurrence risk (HR = 1.271, 95% CI: 1.116–1.447, *p* < 0.001). Patients with recurrent tumors had a 2.741-fold higher risk of recurrence compared to those with primary tumors (HR = 2.741, 95% CI: 1.122–6.697, *p* = 0.027). Tumors located in the extremities carried a 2.62-fold higher recurrence risk than non-extremity tumors (HR = 2.62, 95% CI: 1.15–5.96, *p* = 0.021). The remaining variables, including age, gender, and presence of symptoms, showed no statistical significance in the univariable analysis (all *p* > 0.10). For the significant continuous variable (tumor size) from the univariable analysis, a restricted cubic spline plot visually demonstrated its relationship with the log hazard ratio (**Supplementary Figure S2**), which was approximately linear across the measured range. The linearity assumption test for this variable revealed no significant evidence of nonlinearity (*p* = 0.695), therefore it was treated as a continuous linear term in subsequent models. Given the limited number of recurrence events, the multivariable Cox regression analysis was restricted to a maximum of two covariates to control for overfitting. After adjusting for tumor site, the results identified maximum tumor diameter (per 1-cm increase: HR = 1.283, 95% CI: 1.125–1.463, *p* < 0.001) and extremity location (HR = 2.899, 95% CI: 1.274–6.598, *p* = 0.011) as independent risk factors for postoperative recurrence.

To assess the impact of multiple comparisons on type I error risk, a sensitivity analysis using the Bonferroni correction was performed. As the final model contained two variables, the adjusted significance threshold was set at αadj = 0.025. The *p*-values for both tumor size (*p* < 0.001) and extremity location (*p* = 0.011) were below this stringent threshold, indicating that the significance of these two prognostic factors in the multivariable model was robust.

## 4 Discussion

Although contemporary management of DTs increasingly prioritizes multidisciplinary collaboration and conservative approaches, local resection remains a viable therapeutic option for patients with rapidly progressive or severely symptomatic disease. Our retrospective cohort analysis of 81 DT patients confirmed that tumor location in the extremities, recurrent status, and tumor size (≥7 cm) were independent predictors of postoperative recurrence (all *p* < 0.05). During a median follow-up of 55 months, no patient mortality was observed, and the 28.4% recurrence rate fell within the reported literature range for postoperative DTs recurrence (20%-80%) (16, 17), Recurrence events in this study predominantly clustered within the early postoperative period (≤20 months), with the recurrence curve reaching a plateau after 60 months. Primary DT patients exhibited favorable long-term survival outcomes, achieving a 5-year RFS rate exceeding 70%. Particular vigilance is warranted for the elevated recurrence risk during the first 2 postoperative years, necessitating intensified surveillance within this critical window. Notably, nearly 50% of the cohort were women of reproductive age, suggesting a potential association with hormonal factors. Although hormonal therapies have been empirically used in DTs, they are not currently recommended for routine clinical use due to evidence derived from low-quality studies with limited robust data (6, 8).

### 4.1 High-risk site

Multiple studies suggest that extra-abdominal desmoid tumors, particularly those located in the extremities, may portend a less favorable prognosis (9, 11, 17–20), our findings further substantiate this observation: extremity tumors demonstrated significantly higher recurrence risk compared to non-extremity tumors (HR = 2.899, 95% CI: 1.274–6.598, *p* = 0.011). In the large prospective study by Penel et al. (9), a refined anatomical classification revealed significantly superior 2-year event-free survival (EFS) in tumors at ‘favorable sites’ (abdominal wall, intra-abdominal, breast, digestive viscera, and lower extremities) compared to those at ‘unfavorable sites’ (chest wall, head and neck, and upper extremities) (HR = 0.51, *p* < 0.0001). Within the predictive model developed by Crago et al. (10), not only was the poor prognosis of extremity tumors confirmed, but adjuvant radiotherapy in this subgroup was further associated with an approximately 15% absolute risk reduction. The extremities region has complex anatomy with a high concentration of functionally critical structures such as tendons, neurovascular bundles, and joints. During surgical intervention in this area, attempts to achieve wide resection (R0 margin) carry a substantial risk of damaging these structures, leading to severe postoperative functional impairment—including compromised walking, joint mobility, or overall limb function. Consequently, surgeons often prioritize functional preservation, which may result in inadequate margins (R1/R2) and consequently increase the risk of local recurrence. As anatomical sites most frequently subjected to sustained mechanical stress, extremities are exposed to biomechanical stimuli that may activate the Wnt/β-catenin pathway within tumor cells or potentiate residual tumor cell proliferation through mechanotransduction signaling (21). Moreover, differential aggressiveness across tumor locations may also be attributable to microenvironmental regulation of pivotal signaling pathways that ultimately govern biological aggressiveness (18, 22).

Due to the distinct biological characteristics of DTs in the extremities or concerns among surgeons over potential damage to functional structures, conservative management (such as observation or non-surgical systemic therapy) is often the preferred initial approach for tumors in this region. Even when surgery is performed, the high recurrence rate remains a concern. Therefore, close postoperative monitoring of disease progression and the implementation of a well-structured follow-up protocol are essential.

Furthermore, in the present study, the abdominal subgroup exhibited an extremely low number of recurrence events (2/27), resulting in unstable survival estimates. Therefore, it was not statistically compared as an independent group but was combined with the head/neck and trunk subgroup into a “non-extremity” category for analysis. While this merging may obscure potential prognostic differences among distinct anatomical sites within the abdomen region, it ensures the stability of the statistical model and the clarity of the primary conclusions. Future studies with larger sample sizes are needed to further clarify the distinct biological behavior and prognostic profile of tumors in these locations.

### 4.2 Tumor size and surgical margins

Corroborating multiple prior investigations (10, 17, 18), this study further substantiates that larger tumor size constitutes an independent adverse prognostic factor in DTs (per 1-cm increase: HR = 1.283, 95% CI: 1.125–1.463, *p* < 0.001). However, the clinical utility of tumor size as an independent prognosticator and optimal cutoff determination remain contentious (9, 16, 23). A recent retrospective investigation (15) that employed the median tumor size (6 cm) as the cutoff identified larger tumors as an independent risk factor for DT recurrence (HR = 4.2, 95% CI: 1.8–9.6). Nevertheless, significant heterogeneity persists across studies utilizing identical grouping methodologies (10, 12, 16, 24). DTs exhibit locally aggressive behavior, posing challenges for precise measurement. Significant discrepancies may arise across different operators or methods, with some studies defining tumor size by the maximum diameter recorded in pathological reports (12, 17), while others incorporate preoperative imaging measurements (9, 16). Furthermore, an individual patient data meta-analysis of 7 studies involving DTs treated with resection alone by Timbergen et al. (15) identified a significant association between tumor size and CTNNB1 mutation, and the authors suggested that combining mutational status and tumor size is necessary for predicting recurrence risk. Larger tumor size is generally associated with more aggressive growth, increased surgical difficulty, and more complex anatomical relationships, leading to a higher risk of recurrence. Therefore, for large lesions, watchful waiting or pharmacotherapy may be considered to reduce tumor volume or serve as adjuvant therapy to decrease the complexity of subsequent surgery. During surgery, emphasis should be placed on preserving function and avoiding excessive resection, followed by enhanced imaging surveillance postoperatively. As a continuous variable, tumor size is a reliable predictor and should be incorporated into individualized risk models to achieve precise management and risk stratification.

In this study, the pathological reports did not record margin status, precluding analysis of its prognostic impact. Surgical margin status is a well-established prognostic factor for recurrence in desmoid tumors, with multiple studies confirming that positive or close margins are associated with an increased risk of recurrence (11, 25, 26). Due to the lack of systematically quantified records on margin-to-tumor distance in this study, this variable could not be included in the multivariable model. If larger tumor size or extremity location were associated with a higher likelihood of achieving inadequate margins, then the effect estimates for “tumor size” and “site” in our model might partly reflect the unmeasured confounding influence of margin status (i.e., residual confounding or proxy bias). Consequently, although our findings suggest these are independent factors, their true independence requires further confirmation in future studies with more complete and standardized margin data. However, literature review indicates that the influence of margin status on recurrence risk remains controversial (8–10, 12). A meta-analysis incorporating 16 studies and 1295 patients demonstrates that patients with R1 margins had a 1.78-fold higher risk of recurrence compared to those with R0 margins (11), highlighting the importance of negative margins. However, the nationwide prospective cohort study by the French Sarcoma Group (9), finds that R0 margins yield only a numerical advantage in event-free survival versus R1/R2 margins without statistical significance; furthermore, a retrospective study of 426 desmoid tumor patients reveals significantly inferior progression-free survival (PFS) with R2 versus R0/R1 resection, but no significant PFS difference between R0 and R1 resection (12). However, the traditional R0/R1 dichotomy may have limitations. Cates and Stricker (27) demonstrate that a positive surgical margin or a margin <1 mm is an independent predictor of local recurrence (HR = 9.52, *p* = 0.028). This finding suggests that the traditional R0/R1 classification may inadequately reflect the true risk of recurrence, as the actual recurrence rate is more critically dependent on the precise margin distance. Furthermore, the importance of margin status may be relatively lower compared to function preservation; in the 2023 consensus conference convened by the Desmoid Tumor Working Group (DTWG), experts recommend that for patients with DTs presenting with severe complications, surgery be a necessary intervention, but R0 resection should not be pursued (7). Notwithstanding persistent controversies, this study advocates for the inclusion of tumor size and anatomic location in the prognostic assessment framework for DTs. When planning surgical intervention for high-risk recurrent patients with symptomatic or progressive disease, integration of radiotherapy, local ablation, or systemic therapy warrants deliberate consideration (28–31).

### 4.3 Surgical futility in recurrent desmoid tumor management

In the present study, the univariable analysis revealed a significantly increased risk of recurrence in patients with recurrent disease compared to those with primary tumors (HR = 2.741, 95% CI: 1.127–6.729, *p* = 0.026). Half of the patients in the recurrence group (n=14) experienced postoperative recurrence. Kaplan-Meier survival analysis demonstrated that the recurrence-free survival curve for this group was consistently lower than that of the primary diagnosis group (n=67), indicating a significantly worse prognosis. Some mechanisms may collectively contribute to the poorer prognosis in recurrent patients: Initial treatment potentially “selects for” aggressive tumor subclone; prior therapies disrupt local anatomical barriers, resulting in poorly defined tumor margins upon recurrence, and postoperative adhesions and altered neurovascular anatomy hinder complete resection for fear of iatrogenic injury. Given that postoperative recurrence risk is significantly elevated in recurrent patients, and repeated surgeries may cause functional impairment and cosmetic defects, recurrent patients should consider sorafenib (28) or γ-secretase inhibitors (29) as alternative options. Notably, cryoablation as a local treatment method demonstrates significant potential: in retrospective studies, it shows favorable local control rates, it is especially suitable for high-risk surgical patients (32, 33); it also showed good efficacy in a prospective phase II trial (34), and was highly recognized (7). Therefore, for patients with prior recurrence, the benefit-risk profile of re-operation should be carefully evaluated within a multidisciplinary framework. Alternative strategies, such as systemic therapy, radiotherapy, or local ablation, should be prioritized to achieve disease control while minimizing functional impairment and preserving quality of life.

### 4.4 Radiotherapy in DT management: evidence and controversies

This study found no significant correlation between postoperative radiotherapy and RFS. The therapeutic role of radiotherapy and its optimal clinical application in DTs management remain incompletely defined. However, some studies have suggested that radiotherapy—either as monotherapy or in combination—could potentially provide superior disease control compared to surgery alone (19, 23, 30). The meta-analysis by Janssen et al. (11) demonstrated that radiotherapy significantly reduces postoperative recurrence rates in patients with R1/R2 margins, supporting its role as an adjuvant strategy for incompletely resected cases. However, it is noteworthy that a systematic review of 37 studies indicated adjuvant radiotherapy demonstrated significant superiority specifically only in recurrent cases (*p* < 0.001) (19). The systematic analysis by Smith et al. (35) reported local control rates of 65-83% with radiotherapy alone, whereas combined therapy showed no significant clinical advantage over radiotherapy alone. Retrospective series have demonstrated favorable local control rates with radiotherapy alone (8), a finding further supported by a multicenter prospective phase II trial conducted by the European Organization for Research and Treatment of Cancer (EORTC). In this trial, radiotherapy alone achieved a 3-year local control rate of 81.5% with low rates of severe toxicities (36). Notably, the efficacy of radiotherapy may be modulated by tumor anatomical location and patient age (10, 23, 31). Due to the relatively poor efficacy of radiotherapy in children and young patients and the presence of a risk of secondary malignancies, radiotherapy should be applied cautiously in this population (7, 8, 30). Current international consensus and guidelines indicate that the research evidence supporting adjuvant radiotherapy or radiotherapy alone is “very low”, and radiotherapy can serve as an alternative approach for patients with lesions that cannot be surgically resected or those with positive resection margins (2, 3).

This study has several limitations. First, as a single-center retrospective analysis, the follow-up strategies were not entirely uniform across the observation period. Although we systematically reviewed all imaging and clinical records and applied a unified, objectively evidence-based definition of recurrence (new lesion confirmed by imaging or pathology) to ensure endpoint consistency as much as possible, variation in follow-up remains an inherent limitation of the retrospective design. Second, a significant limitation is the absence of data on surgical margin status (R0/R1). During the study period, standardized circumferential margin assessment was not part of the routine pathological workflow for this rare disease at our institution. Since margin status is a well-established key prognostic factor, its absence may introduce residual confounding in the multivariable model. Specifically, if larger tumor size or specific anatomical locations (e.g., extremity) were more frequently associated with positive margins, the HR for tumor size and site reported in our study might be overestimated, as their effects could partly reflect the influence of this unmeasured factor. Therefore, the interpretation of tumor size and location as completely independent prognostic factors requires caution.

Notwithstanding these limitations, this study accurately reflects the treatment course and outcomes for desmoid tumors within a specific historical and clinical context, and the provided real-world evidence retains value. The aforementioned limitations also clearly indicate directions for future research: there is a need for prospective studies that incorporate standardized pathological margin assessment protocols and uniform active surveillance follow-up plans as core design elements to more precisely delineate the independent prognostic value of various factors.

## 5 Conclusion

Our study confirms that larger tumor size and extremity location are independent predictors of worse RFS in DTs. Based on these core high-risk factors, we recommend a multidisciplinary, risk-adapted management strategy: For large or extremity DTs, initial active surveillance or neoadjuvant/systemic therapy (e.g., γ-secretase inhibitors for progressing cases) should be prioritized over immediate radical resection when function is at risk, complemented by CTNNB1 testing for risk stratification. Even after radical surgery, the postoperative recurrence rate remains considerable, particularly in patients with prior recurrence. Current evidence indicates that radical resection is no longer the universal standard; systemic therapy or ablation are effective alternatives, though surgery retains a role in symptom control for abdominal/abdominal wall tumors. Future studies integrating biomarker profiling are needed to elucidate molecular heterogeneity and optimize multimodal regimens for high-risk patients.

## Data Availability

The original contributions presented in the study are included in the article/**Supplementary Material**. Further inquiries can be directed to the corresponding author.

## Supplementary material

Supplementary Figure 1 Exploratory analysis of RFS by tumor location. Kaplan-Meier curves show RFS for the initial three-category classification of tumor location (Head & Neck/Trunk, Extremity, Abdomen).

Supplementary Figure 2 Restricted cubic spline analysis of the association between tumor size and recurrence risk. The plot illustrates the continuous relationship between tumor size (in centimeters) and hazard ratio for recurrence, modeled using a restricted cubic spline with three knots. The solid orange line represents the hazard ratio, with the gray shaded area indicating the 95% confidence interval. The non−linear association was not statistically significant (*p* for linear hypothesis = 0.695). Axes: X−axis, tumor size (cm); Y−axis, hazard ratio.
