# Supplementary figures and images for: Risk factors for postoperative recurrence of desmoid tumors: a retrospective cohort analysis
Source: Front Oncol. 2026 Jan 16;15:1677325. doi: 10.3389/fonc.2025.1677325 (PMC12856494; doi:10.3389/fonc.2025.1677325)

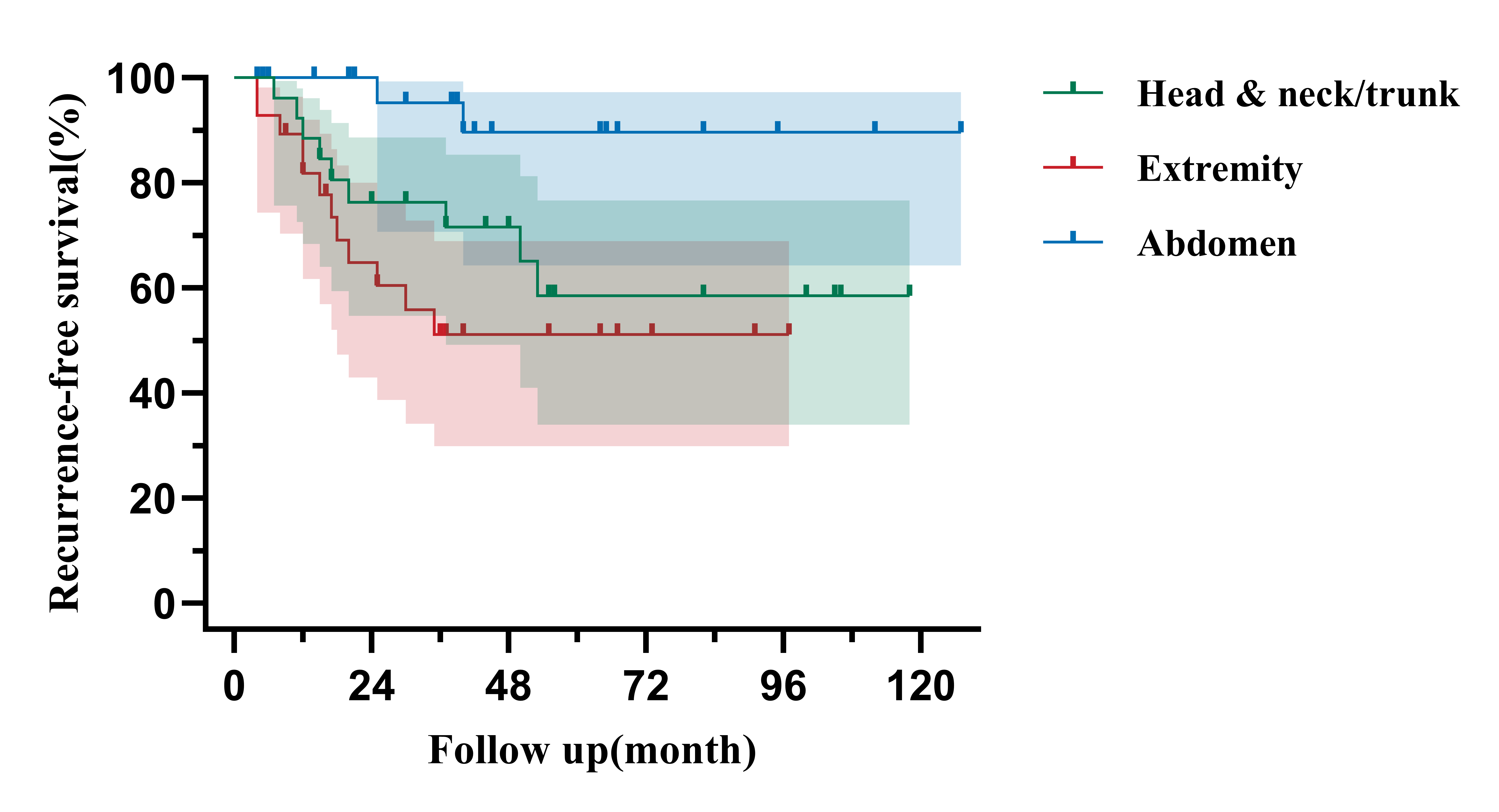

Supplement: Supplementary Figure 1 — Exploratory analysis of RFS by tumor location. Kaplan-Meier curves show RFS for the initial three-category classification of tumor location (Head & Neck/Trunk, Extremity, Abdomen). [file Image1.tif]

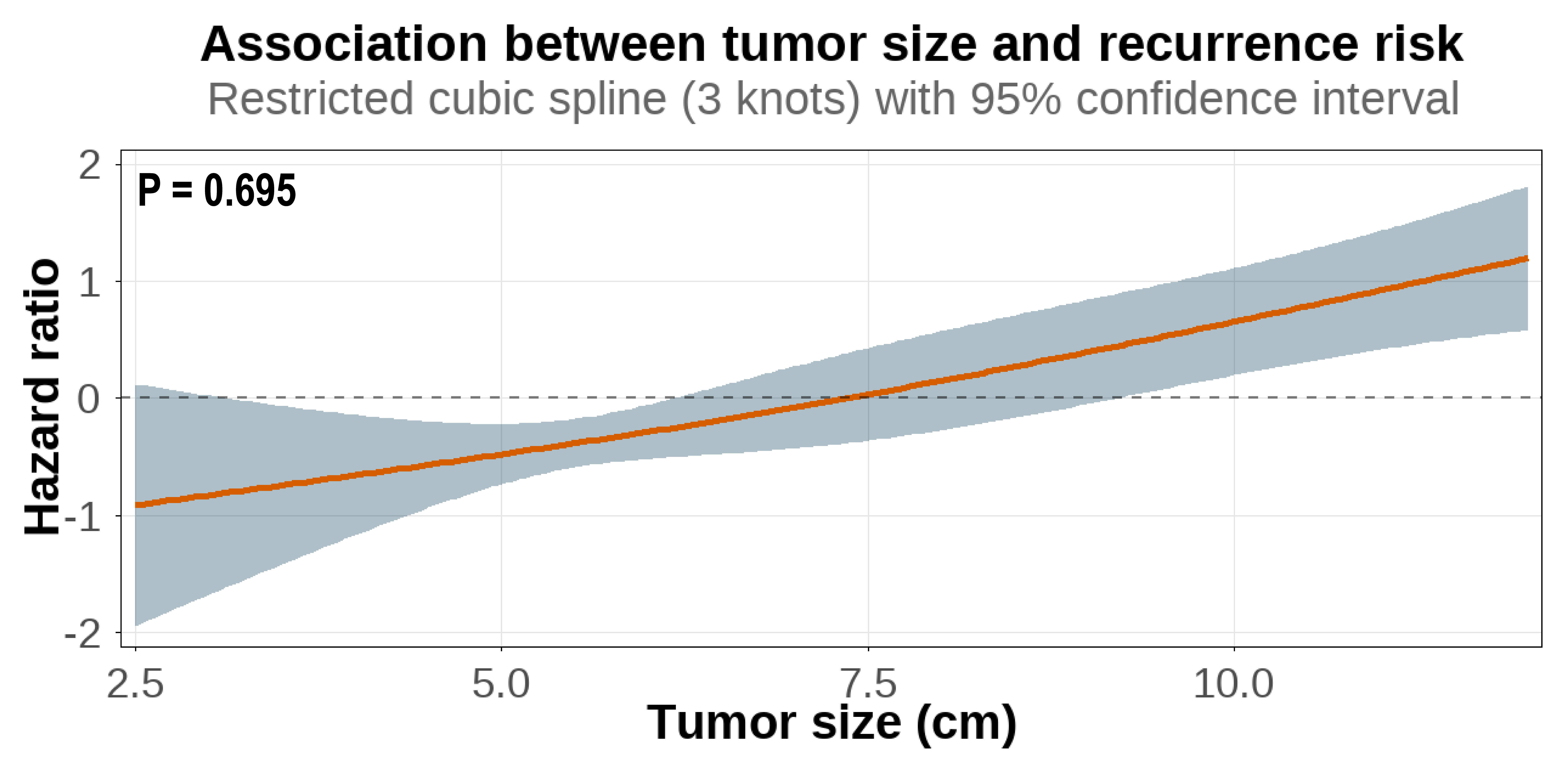

Supplement: Supplementary Figure 2 — Restricted cubic spline analysis of the association between tumor size and recurrence risk. The plot illustrates the continuous relationship between tumor size (in centimeters) and hazard ratio for recurrence, modeled using a restricted cubic spline with three knots. The solid orange line represents the hazard ratio, with the gray shaded area indicating the 95% confidence interval. The non−linear association was not statistically significant (p for linear hypothesis = 0.695). Axes: X−axis, tumor size (cm); Y−axis, hazard ratio. [file Image2.tif]
